# Optimization of a Methodology for Quantification and Removal of Zinc Gives Insights Into the Effect of This Metal on the Stability and Function of the Zinc-Binding Co-chaperone Ydj1

**DOI:** 10.3389/fchem.2019.00416

**Published:** 2019-06-11

**Authors:** Jemmyson Romário de Jesus, Annelize Zambon Barbosa Aragão, Marco Aurélio Zezzi Arruda, Carlos H. I. Ramos

**Affiliations:** ^1^Department of Organic Chemistry, Institute of Chemistry, University of Campinas – UNICAMP, Campinas, Brazil; ^2^National Institute of Science and Technology for Bioanalytics, Institute of Chemistry, University of Campinas – UNICAMP, Campinas, Brazil; ^3^Spectrometry, Sample Preparation and Mechanization Group – GEPAM, Institute of Chemistry, University of Campinas – UNICAMP, Campinas, Brazil; ^4^National Institute of Science and Technology for Bioimage and Structural Biology (INBEB), Federal University of Rio de Janeiro (UFRJ), Rio de Janeiro, Brazil

**Keywords:** ICP-MS, zinc, Hsp40, protein folding and stability, protein structure/function relationship, homeostasis

## Abstract

Ydj1, a class B J-protein (Hsp40) in yeast, has two zinc finger domains in each monomer and belongs to an important co-chaperone family that plays crucial roles in cells, such as recognizing and binding partially folded proteins and assisting the Hsp70 chaperone family in protein folding. Yeast cells with *ydj1* deletion were less efficient at coping with zinc stress than wild-type cells, and site-directed mutagenesis studies that impair or delete the zinc finger region have confirmed the importance of this region to the function of Ydj1; however, little is known about whether the presence of zinc is critical for the function of the protein. To gain insights into the effect of zinc on the structure and function of Ydj1 without having to modify its primary structure, a method was developed and optimized to quantify and remove the zinc from the protein. Recombinant Ydj1 was produced and purified, and its zinc content was determined by ICP-MS. The result showed that two zinc atoms were bound per monomer of protein, a good indicator that all sites were saturated. To optimize the removal of the bound zinc, variations on chelating agent (EDTA, EGTA, 1,10-phenanthroline), chelator concentration, reaction time, pH, and temperature were tested. These procedures had no effect on the overall secondary structure of the protein, since no significant changes in the circular dichroism spectrum were observed. The most significant removal (91 ± 2%, *n* = 3) of zinc was achieved using 1,10-phenanthroline (1 × 10^−3^ mol L^−1^) at 37°C with a pH 8.5 for 24 h. Zinc removal affected the stability of the protein, as observed by a thermal-induced unfolding assay showing that the temperature at the middle of the transition (Tm) decreased from 63 ± 1°C to 60 ± 1°C after Zn extraction. In addition, the effect on the ability of Ydj1 to protect a model protein (luciferase) against aggregation was completely abolished after the Zn removal procedure. The main conclusion is that zinc plays an important role in the stability and activity of Ydj1. Additionally, the results highlight the medical importance of chaperones, as altered zinc homeostasis is implicated in many diseases, such as neurodegenerative disorders.

## Introduction

There is mounting evidence that the homeostasis of metals has an important medicinal role, since the disturbance of this equilibrium appears to be related to the occurrence of severe diseases and to aging (Larbi et al., 2006; Susuki et al., 2008; Montes et al., 2014). The dietary supplementation of zinc, and to a lesser extent copper, seem to alleviate the symptoms of neurodegenerative diseases and dementia (Kawahara et al., 2014; Tsunemi and Krainc, 2014; Tanaka and Kawahara, 2017). Curiously, there is also mounting evidence that molecular chaperones and heat shock proteins (Hsps) participate in metal homeostasis, since their expression increases when metals are added to the cellular environment (Larbi et al., 2006; Pirev et al., 2009; Tiffany-castiglioni and Qian, 2012;Singla and Dhawan, 2016).

**Figure 1:**
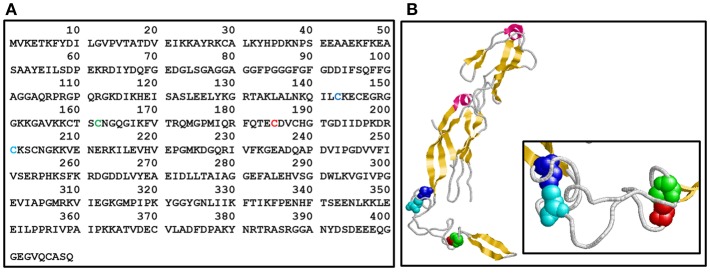
Ydj1 structure. **(A)** Primary structure. Amino acid sequence of Ydj1 (UniProtKB - P25491) from *Saccharomyces cerevisiae*. **(B)** Tertiary structure. Crystal structure of the Ydj1 monomer (residues 110-337; PDB 1NLT). The Cys residues in each Zn binding domain (ZBD) are shown in a space-filling model: Cys143 (blue) and Cys-201 (cyan) in ZBDI and Cys162 (green) and Cys185 (red) in ZBDII [the same colors are also used in **(A)**]. Pink, alpha-helix; yellow, strand.

Molecular chaperones and Hsps are expressed to cope with protein aggregation, a phenomenon that results from the crowded environment inside the cell (Wong and Houry, 2004; Tiroli-Cepeda and Ramos, 2011; Lindberg et al., 2015). Therefore, these proteins help to maintain homeostasis during regular and stressful situations and may be divided into foldases (Hsp70 and Hsp90, for instance) and holders, which bind partially unfolded proteins to protect against aggregation (e.g., Hsp40s and small Hsps). One important protein from the Hsp40/J-protein family (Cyr and Ramos, 2015; Kampinga et al., 2018) is Ydj1, which is a cytosolic protein from yeast. Ydj1 contains a conserved zinc finger-like region (named ZFLR, residues 143-209) that has two zinc-binding domains (named ZBDI and ZBDII) (Figure 1) formed by four repeats of the Cys-X-X-Cys-X-Gly-X-Gly motif (Li et al., 2003; Fan et al., 2005), which is characteristic of class A Hsp40/J-proteins (Bardwell et al., 1986; Martinez-Yamout et al., 2000). Cys-201 and Cys143 in ZBDI and Cys162 and Cys185 in ZBDII (Figure 1) participate in the tetrahedral coordination of zinc (Fan et al., 2003).

Studies aimed at understanding the influence of zinc in the function of class B J-proteins, such as Ydj1, have used site-directed mutagenesis to disrupt one or more zinc binding sites (e.g., Linke et al., 2003; Fan et al., 2005). Although a large amount of knowledge was obtained from these works, we wondered whether it was possible to develop a method that allowed for efficient zinc extraction without perturbing the wild-type sequence of Ydj1. This method is described here, and its effects on the structure and function of Ydj1 are discussed. The method proved to be very efficient, and we are confident that it can be put to good use by researchers investigating other proteins that bind zinc.

## Materials and Methods

### Reagents, Standards, and Protein

Sub-boiled nitric acid (HNO_3_, 69% w/v, Merck) was prepared by sub-boiling distillation in quartz stills. Hydrogen peroxide (H_2_O_2_) and luciferase (client protein) were purchased from Sigma Aldrich. Certified reference material from the Institute for Reference Materials and Measurements (IRMM-3702) was used to evaluate method performance. Ultra-pure water with >18 MΩ, resistivity, obtained using a Milli-Q high purity water system (Millipore, USA), was used for dilution of standards, preparing samples and final rinsing of the acid-cleaned vessels. All containers were decontaminated in HNO_3_ 10% (v/v) prior to use. A stock standard solution of Zn was prepared by dissolving metal in sub-boiled acids. The working standard was prepared at different concentrations by diluting the stock solution to 1 mg L^−1^. Polypropylene 50-mL centrifuge tubes were used for sonication experiments. Recombinant Ydj1 was produced as previously described (Silva et al., 2011).

### Zinc Tolerance Assay Using *S. cerevisiae*

The following yeast strains were used in this study: BY4741 (MATa his3Δ1 leu2Δ0 met15Δ0 ura3Δ0) and an isogenic *ydj1* knockout (Δ*ydj1*) that was kindly provided by Dr. Claudio Akio Masuda (Federal University of Rio de Janeiro—UFRJ). The yeast strains were grown using liquid or solid YPD (yeast peptone dextrose) for maintenance and were transformed by the LiAc/PEG method (Elble, 1992) with an empty vector (SM640, low copy, URA3 marker, and GAL promoter). The zinc tolerance assay was performed using the transformed yeasts, selected by growth in synthetic complete solid media lacking uracil (SC-U), in the absence and presence of increasing concentrations of ZnSO_4_ (0.5–5.0 mmol L^−1^). Overnight cultures were centrifuged, and the pellets were resuspended into 40 mL of SG-U liquid to make a starter culture (A_600nm_ = 0.10). The cells were grown to 0.6–0.8 at 30°C under constant agitation. After the desired growth was reached, serial dilutions (10-fold each) were made in triplicate using a 96-well-plate, and 2.5 μL from each dilution was spotted onto SG-U plates in the absence and presence of increasing concentrations of ZnSO_4_ (0.5–5.0 mmol L^−1^). Plates were incubated at 30°C for a period of 72 h and colonies were manually counted to determine growth restriction.

### Protein Purification

Ydj1 was expressed in *Escherichia coli* BL21(DE3)pLys by induction with 0.4 mmol L^−1^ isopropyl thio-b-D-galactoside (IPTG). The cells were lysed in 50 mmol L^−1^ Tris-HCl (pH 8.0), 500 mmol L^−1^ KCl and 1 mmol L^−1^ EDTA buffer (15 mL per L of LB) and centrifuged 60 min at 26,000 × g. The protein was purified using two chromatographic steps as previously described (Silva et al., 2011) with few modifications. Briefly, Ydj1 was subjected to anionic chromatography on a Macro-Prep(R) High Q Support Resin (BioRad) using an ÄKTA FPLC instrument (Pharmacia Biotech) equilibrated with 20 mmol L^−1^ phosphate (pH 7.5) and eluted by a NaCl gradient (final concentration was 500 mM). Further purification was performed by size exclusion chromatography using a Superdex 200 pg column on an ÄKTA FPLC (Pharmacia Biotech) previously equilibrated with 25 mmol L^−1^ Tris-HCl buffer (pH 7.5) and 500 mmol L^−1^ NaCl (unless otherwise stated, all experiments were conducted using this buffer). The efficacy of the purification was checked by 12% (v/v) SDS-PAGE.

### Circular Dichroism Analysis

Circular dichroism (CD) experiments were measured in a Jasco J-810 spectropolarimeter coupled to a Peltier-type System PFD 425S for temperature control. Spectra were collected at 4°C from 200 to 260 nm at a scan rate of 20 nm min^−1^ with a spectral bandwidth of 1 nm. Starting protein concentration was 20 μmol L^−1^ using a cell with a 0.2-mm path length. Each spectrum was the average of at least eight scans, which corrected against a buffer blank. Thermal stability was monitored at 222 nm by heating in increments of 1°C min^−1^ in a cell with a 0.2-mm path length and a spectral bandwidth of 1 nm. Each curve was the average of at least three independent experiments.

### Zinc Extraction Procedure

**Figure 2:**
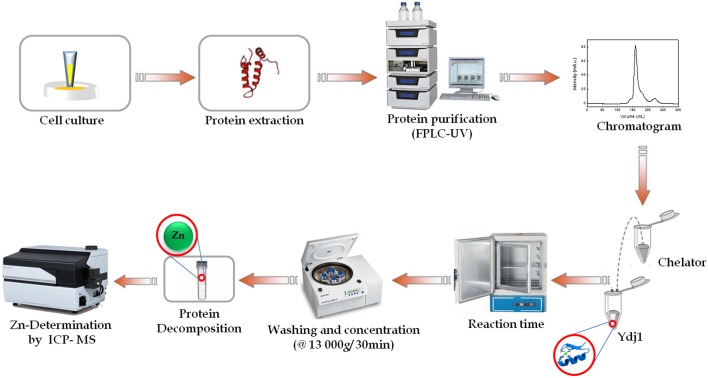
Schematic flow of this work. General schematic of the expression and purification of Ydj1, as well as the zinc removal procedure. First, cell culturing was performed to express Ydj1. Then, Ydj1 was isolated free of contaminants using liquid chromatography. Then, an optimization methodology was performed to remove Zn from Ydj1. ICP-MS was used for Zn quantification analysis and to evaluate the efficiency of Zn removal.

Experiments with zinc were conducted in batch, and extraction experiments were tested with different chelating agents (1,10-phenantroline, EDTA and EGTA) at various concentrations (1.10^−3^-1.10^−2^ mmol L^−1^), pH levels (7.5–9), incubation temperatures (4–37°C) and extraction times (1–48 h). A protein concentration of ~2 μmol L^−1^ was used for each extraction. All pH adjustments were performed using a NaOH solution. All experiments were conducted in triplicate, and the protein conditions were monitored by CD. At the end of the designated incubation period, the slurries were washed and concentrated using a Centricon tube, filtered through a 0.2-μm Whatman membrane filter. Filtrates were then decomposed and analyzed using inductively coupled plasma-mass spectrometry (ICP-MS). Figure 2 presents a general scheme of Ydj1 expression and purification, as well the Zn removal procedure using optimal conditions (see the Results and Discussion sections for details).

### Sample Preparation

Samples were decomposed using a sonicator ultrasonic processor Qsonica® (Newtown, USA), consisting of a cup-horn shaped sonotrode, which can accommodate up to eight Eppendorf vials, for ultrasound-assisted decomposition. First, ~250 μL of Ydj1 solution (final concentration 7 μM) was transferred to Lobind microtubes containing the extracting solution composed of 250 μL HNO_3_ (40% v/v) + H_2_O_2_ (30% v/v). Then, the mixture (sample + extracting solution) was sonicated at room temperature for 8 min with a power of 210 W. After sonication, the liquid supernatant was separated from the solid phase by centrifugation for ~10 min at 10,000 × g. The supernatant was then transferred to a clean and bulged Falcon tube, and 1.5 mL was analyzed by ICP-MS. Blank solutions were prepared in the same way as the test samples and were measured for each extraction series. The measurements were performed in triplicate for each extraction procedure, using an external calibration method. Therefore, the metal content in each sample was based on the calibration curve obtained from the standards. Finally, the extraction efficiency of Zn after ultrasonic extraction was calculated using the following equation:

(1) $$\begin{array}{l} Extraction\;efficiency\;(\%)=\left(\frac{true\;value-obtained\;value}{true\;value}\right)\\ \;\times \;100 \end{array}$$

### Determination of Zinc by ICP-MS

Zn quantification was performed using an inductively coupled plasma mass spectrometer (ICP-MS) from Shimadzu (model 2030) equipped with an octapole analyzer, mini-torch and a concentric nebulizer. The nebulization process was carried out at a constant temperature (5°C) and the separation of the ions in the analyzer was performed using a collision cell filled with helium gas (He). The voltage settings of the ion lens as well as other parameters of the instrument were checked daily with solution containing beryllium (Be, 10 μg L^−1^), indium, bismuth, cerium (In, Bi, Ce, 2 μg L^−1^), cobalt and manganese (Co and Mn, 5 μg L^−1^). The nebulizer gas, auxiliary gas, plasma gas (mini-torch), and cell gas flows were 0.7, 1.1, 8.0, and 6.0 L min^−1^, respectively. Lens voltage was −21 V and the energy filter was 7.0 V. Analytical curves covered Zn concentrations from 5 to 100 μg L^−1^.

### Isothermal Titration Calorimetry

Isothermal titration calorimetric (ITC) experiments were carried out on a MicroCal VP-ITC unit (MicroCal, Inc., Northampton, MA) at 25°C. Solutions of titrants and titrates were made from the same buffer [25 mmol L^−1^ Tris-HCl buffer (pH 7.5) and 500 mmol L^−1^ NaCl] and thoroughly degassed prior to use. Each ITC experiment consisted of 3 μl injections of either 2, 3, or 4 mM ZnCl_2_ to 30 μM Ydj1 (monomer concentration) with an interval of 300 s between injections. The data were analyzed by Microcal Origin software using the one-set-of-sites model to calculate the apparent enthalpy change (ΔH_app_), which is measured directly, stoichiometry (n), apparent association constant (K_Aapp_), and apparent entropy change (ΔS_app_). The heat of the ligand dilution was determined from its titration into the buffer solution and from the end of titration curve into the protein receptor and subtracted of the data.

### Chaperone Activity

Chaperone activity was studied by measuring the ability of Ydj1 to prevent the aggregation of luciferase (a client protein) as previously described (Borges et al., 2005; Tiroli and Ramos, 2007) with little modification. Briefly, a 5-μL aliquot of luciferase (10 mg mL^−1^, Sigma Aldrich) was diluted 100-fold into buffer (25 mM Tris-HCl, pH 7.5, 500 mM NaCl) to a final concentration of 3 μM. Then, luciferase (3 μM) was incubated at 37°C for 60 min in the absence and presence of Ydj1 before and after the Zn removal procedure. Aggregation was assayed by measuring light scattering (turbidity) at 320 nm using a fluorescence spectrophotometer (Varian Cary Eclipse), and the total aggregation of luciferase was set as standard (100%). The percent protection was obtained using the following equation:

(2) Protection (%)=(Δe  Δech)x 100

where Δe is the light scattering measurement for luciferase alone after 60 min, and Δe_ch_ is each of the other measurements at the same point.

### Statistical Analysis

All the values were represented as the means and relative standard deviation after analyses in triplicate. ANOVA software (Xia and Wishart, 2016) was used to analyze the significant differences between the groups. The *p* < 0.05 were considered statistically significant.

## Results and Discussion

### Ydj1 Helps Yeast Cells Tolerate Increasing Concentrations of Zinc

Exposure to increasing concentrations of metals is related to an increase in stress and toxicity in cells (Valko et al., 2005). One of the several strategies that cells use to deal with stress is the expression of a family of proteins known as chaperones and Hsps (Tiroli-Cepeda and Ramos, 2011). One of these proteins, which belongs to the class A J-domain proteins in yeast, is named Ydj1 (Fan et al., 2005). Figure S1 shows that the growth of wild-type yeast cells was not perturbed by the presence of up to 2 mmol L^−1^ Zn; however, 5 mmol L^−1^ Zn decreased cell survival by approximately one-fourth. This effect was even worse in yeast cells with a deleted *ydj1* gene, as even 0.5 mmol L^−1^ of Zn caused inhibition, which seemed to be enhanced when 5 mmol L^−1^ of Zn was added (Figure S1).

**Figure 3:**
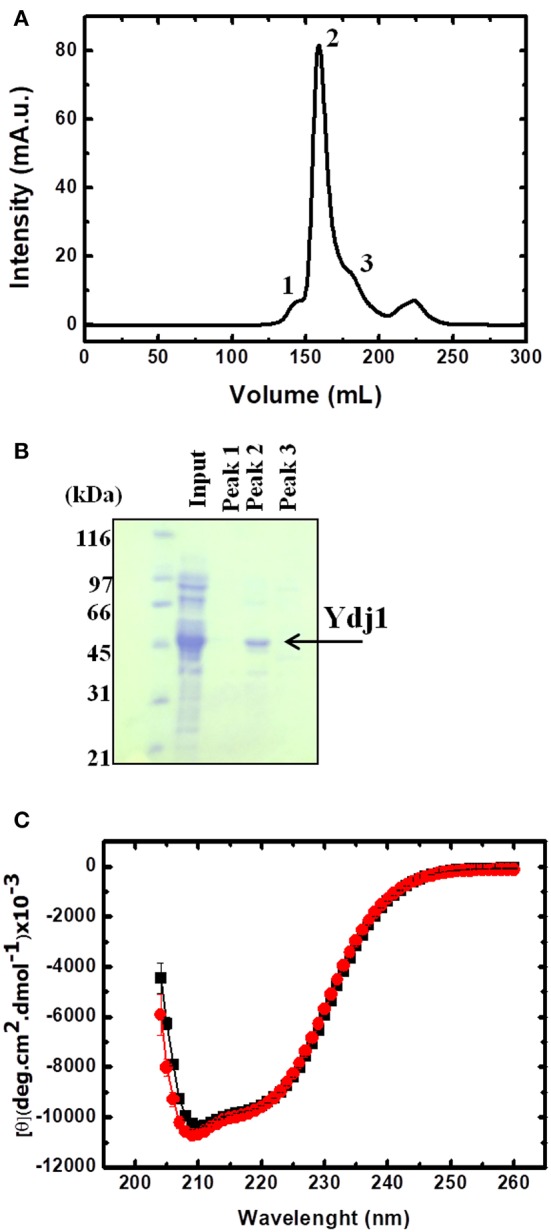
Production and purification of folded Ydj1. **(A)** Chromatogram of the last purification step. Peaks were labeled 1 to 3 and verified by SDS-PAGE **(B)**. Sample from peak 2 contained purified Ydj1. Molecular mass standard is on the left. **(C)** Folded Ydj1 was produced, as verified by its far-UV CD spectrum (red). Ydj1 remained folded after the Zn removal procedure (see text for details), as verified by its far-UV CD spectrum (black).

To gain further insight into the effect that Zn has on Ydj1, a recombinant protein was produced and a new and efficient methodology to extract the bound Zn was developed. Figure 3 shows the resultant chromatogram (Figure 3A) and SDS-PAGE gel (Figure 3B) obtained after the purification step. From Figure 3B, it was determined that the protein had a significant purity from ~95% as calculated by ImageJ software (https://imagej.nih.gov/ij/?).

### Ydj1 Was Purified Folded and With 2 mol of Zn Per Monomer

Ydj1 was produced folded as shown by its far-UV CD spectrum profile (red in Figure 3C). The CD spectrum showed a clear minimum at ~208 nm, characteristic of proteins with alpha-helical domains, as is the case of the J-domain in Hsp40 proteins (Fan et al., 2003). The spectrum also had a significant signal between 210 and 220 nm, characteristic of proteins with beta-sheet rich domains, as is the case of the C-terminal domain in Hsp40 proteins (Figure 1; Li et al., 2003). The analysis of the CD spectrum profile indicated a well-folded protein that was very similar to those of other homologs from the Hsp40 family (Ramos et al., 2008; Couturier et al., 2010; Silva et al., 2011).

To evaluate the possible influence of Zn on the structure and functional activity of the Ydj1, it was necessary to determine the concentration of Zn in this co-chaperone. Therefore, ~7 μmol L^−1^ of protein was decomposed and analyzed by ICP-MS, showing a total concentration of ~937 ± 10 μg kg^−1^ (*n* = 3) and resulting in a molar ratio of 1 mol of protein per 2 mols of Zn. These results are in good agreement with the fact that each Ydj1 polypeptide has two zinc-binding domains (ZBDI and ZBDII) (Figure 1) formed by four repeats of the Cys-X-X-Cys-X-Gly-X-Gly motif, which is characteristic of class A J-proteins (Bardwell et al., 1986; Martinez-Yamout et al., 2000). Therefore, one can conclude that the Ydj1 produced in this work binds Zn and that the purification procedure results in a protein in which both ZBDI and ZBDII coordinate one Zn ion each.

### Optimization of the Zinc-Extraction Procedure

#### Influence of Chelating Agent and pH

The extraction of Zn from zinc binding proteins is a powerful strategy for investigation of the effect of Zn on the structure and function of these proteins. Such a strategy is preferred over site-directed mutagenesis, which may cause other unexpected effects such as conformational and stability changes. Therefore, this work aims to develop an optimized method to extract the Zn and to test this method by investigating the effect of Zn removal on the structure and function of a zinc binding protein, Ydj1.

**Figure 4:**
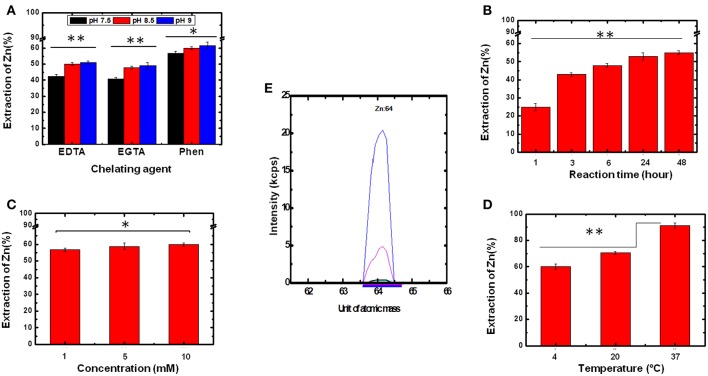
Determination of the optimum conditions for Zn removal. To determine the best experimental parameters for the removal of bound Zn, several conditions were tested. Statistical analyses were performed using ANOVA and considered significant for *p > 0.05 and **p < 0.05. **(A)** Chelating agent and pH. **(B)** Reaction time. **(C)** Chelating agent concentration. **(D)** Temperature. **(E)** ICP-MS spectra of purified Ydj1 before (blue) and after (red) the use of the best conditions for Zn removal (see text for details). Blank control (black).

Initially, three different chelating agents were evaluated, (i) 1,10-phenantroline; (ii) EDTA, and (iii) EGTA, at three different pH levels (7.5, 8.5, and 9.0). The chelating agents were chosen considering their formation constants and the number of binding sites present in each, which strongly depends on pH. Additionally, the range of pH tested considered the maintenance of protein stability. Thus, a final concentration of 1 mmol L^−1^ of each chelating agent was incubated with 2 μmol L^−1^ of Ydj1 solubilized in 500 μL of elution solution (containing 25 mmol L^−1^ Tris-HCl + 500 mmol L^−1^) at three different pH levels (7.5, 8.5, and 9.0) over 48 h. The results, after ICP-MS analysis, are shown in Figure 4. For all chelating agents, the use of pH 8.5 and pH 9.0 were more effective than pH 7.5 in the removal of Zn ions (Figure 4A). The best results for Zn removal were at pH 9.0: the use of EGTA removed 49 ± 1% (*n* = 3), the use of EDTA removed 51 ± 1% (*n* = 3) and the use of 1,10 phenantroline removed 60 ± 2% (*n* = 3) (Figure 4A). These results indicate that pH contributes significantly to the Zn removal procedure, especially when the reaction involves EDTA and EGTA. This is a reasonable observation because, in a basic environment, there is deprotonation of the chelating agents, thus resulting in a more efficient removal of Zn (Lim et al., 2005). The strongest removal was observed when 1,10-phenantroline was used, as expected when its higher ability to form chelate with the metal ions was considered (Pessôa et al., 2011, 2013). Therefore, as the best results were obtained using 1,10-phenanthroline and as there was no significant difference between the results obtained using pH 8.5 and 9.0, 1,10-phenanthroline and pH 8.5 were chosen to optimize the following Zn removal strategies (see below).

#### Influence of Reaction Time

From the results discussed above, further development of the methodology for Zn removal used 1.0 mmol L^−1^ 1,10-phenantroline and pH 8.5. In the next step, the experiments were performed at 4°C to investigate the effect of reaction time on Zn removal (Figure 4B). The results indicated an increase on the extraction efficiency of Zn as reaction time increased from 1 to 48 h. This fact is clearly visible when comparing the result obtained at 1 h with that obtained at 3 h, in which the removal percentage increased from 25 ± 2% to 43 ± 1% (*n* = 3) (Figure 4B). However, between 24 and 48 h, the extraction did not change significantly, as Zn removal increased from 53 ± 2% to 55 ± 1% (*n* = 3). Thus, the reaction time of 24 h was chosen as the best for the aims established for this work. Although no change was observed in the CD spectrum profile of Ydj1, it is wiser, due to their perishable nature, to use a protein incubation period of 24 h rather than 48 h as the longer time added no significant benefit to the results.

#### Influence of Chelating Agent Concentration

For the next step of investigation, the optimized results obtained (pH 8.5, incubation time of 24 h and temperature at 4°C) were kept constant to investigate the effect of the concentration of chelating agent on Zn removal. Concentrations of 1, 5, and 10 mmol L^−1^ were tested but the smallest one was already satisfactory as there was no improvement in removal between the conditions tested (Figure 4C). This result supports the high affinity that Zn has for at least one of the ZBDs of the protein (see further discussion below). Thus, the chelating agent concentration of 1 mmol L^−1^ was used for the next experiments.

#### Influence of Temperature

Finally, the effect of temperature on the extraction of Zn was also investigated. Once again, the optimized results (1.0 mmol L^−1^ 1,10-phenantroline, pH 8.5, and incubation time of 24 h) were kept constant. Temperatures tested were 4, 20, and 37°C (Figure 4D). This parameter was shown to be critical for optimization of the extraction process as Zn removal efficiencies increased as temperature increased from 4 to 37°C: the extraction at 4°C had a removal efficiency of 57 ± 2% (*n* = 3), and at 37°C the removal efficiency was 91 ± 2% (*n* = 3) (Figure 4D). That temperature is an important factor in the removal of metal can be explained in terms of a decrease in the force of binding between the metal and protein caused by the energy generated by heating, thus increasing the competition of chelating agents for the metal. Based on this observation, the temperature of 37°C was fixed as the optimum temperature for Zn extraction. Therefore, the optimized conditions were as follows: 1.0 mmol L^−1^ 1,10-phenantroline, pH 8.5, incubation time of 24 h and temperature at 37°C. The structure and function of Ydj1 were further investigated treated using these conditions (see below).

#### Evaluation of Performance of the Method

The experimental conditions tested and the optimum outcomes obtained by the studies described in this work are shown in Table 1. As described above, the incubation of Ydj1 with 1,10-phenanthroline (1.0 mmol L^−1^) for 24 h at a pH of 8.5 and a temperature of 37°C resulted in the most significant Zn removal (91 ± 2%, *n* = 3), representing a final concentration of 23 ± 2 μg L^−1^ (*n* = 3) (initial = 260 ± 1 μg L^−1^ (*n* = 3). As a final confirmation, the ICP-MS spectrum of purified Ydj1 (blue) was compared with that of Ydj1 after (red) the use of the optimized methodology developed in this study (Figure 2E), confirming a significant removal of Zn. Quantification of the Zn remaining in Ydj1 after the procedure, performed using an external calibration curve, indicated a stoichiometry of <0.2 mol of Zn per mol of a monomer of Ydj1. Detection (LOD) and quantification (LOQ) limits were 0.03 and 0.10 μg L^−1^, respectively. The method proposed was linear over the range tested (5–100 μg L^−1^ with *r*^2^ = 0.9998), and the relative standard deviation was <4% (*n* = 3). The accuracy of the method proposed was checked by the analyses of a certified reference material (IRMM-3702). The results were in good agreement with the certified values.

**Table 1:** Experimental conditions for the extraction of zinc from Ydj1.

**Variable parameter**	**Studied interval**	**Optimum**
Chelating agent	EDTA, EGTA, 1,10-phenantroline	1,10-phenantroline
Medium pH	7.5–9.0	8.5
Incubation time (hours)	1–48	24
Chelating agent concentration (mmol L^−1^)	1–10	1
Incubation temperature (°C)	4–37	37

### On the Structure and Function of Ydj1 After the Zn-Extraction Procedure

**Figure 5:**
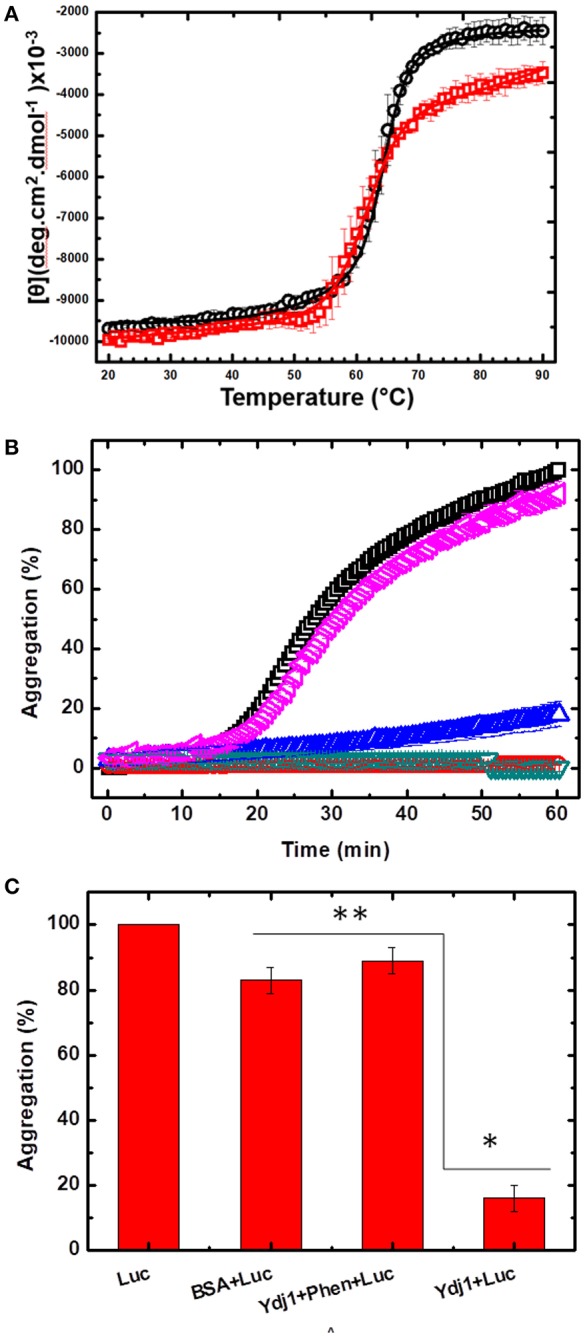
The effect of Zn removal on the stability and chaperone function of Ydj1. **(A)** Heat-induced unfolding was measured by heating Ydj1 before (red squares) and after (black circles) the Zn removal procedure; the samples were heated from 20 to 90°C, and the CD signal was measured at 222 nm. Data were fitted using Origin. **(B)** Chaperone activity. Luciferase aggregation at 37 °C was monitored by light scattering (turbidity) (black square). Ydj1 alone, either before (red inverted triangle) or after (green inverted triangle) the Zn removal process remains folded during the process. Ydj1 before (blue triangle) the Zn removal process protected luciferase against aggregation but did not after (pink inverted triangle) the Zn removal process. **(C)** Bar chart summarizing the protection efficiency of Ydj1 before and after Zn removal, using luciferase as the client protein, against aggregation during heat at 37°C for 60 min [as shown in **(B)**]. *p > 0.05 and ** *p* < 0.05. Bovine serum albumin (BSA) was used as a control, showing that the mere mixture with any polypeptide did not prevent protection against aggregation.

Once the procedure to remove Zn from Ydj1 was optimized, we initiated an investigation of the effects of the extraction on the structure and function of the protein. First, we investigated the effect on the structure of the protein by measuring the far-UV CD spectrum and compared it to that of the protein before the removal procedure (Figure 3C). As discussed above, Ydj1 was produced in a folded conformation (Figure 3C, red spectrum) and remained folded after the Zn removal procedure (Figure 3C, black spectrum). Since the spectra were indistinguishable, one can conclude that Zn removal has no effect on structure. However, modifications that do not affect the structure but do affect stability cannot be discarded. Therefore, we investigated the thermal stability of the protein by heating it up to 90°C and followed the results with measurement of the CD signal at 222 nm (Figure 5A). Purified Ydj1 was stable up to 55°C and unfolded via a transition in which Tm, the temperature at the midpoint, was 60 ± 1°C (*n* = 3). After the Zn removal procedure, Ydj1 showed a similar heat-induced profile but with a Tm equal to 63 ± 1°C (*n* = 3). However, the CD signal after the transition was higher in the sample before Zn removal than after, probably due to aggregation. The heat-induced unfolding results indicated that the removal of Zn had negligible effects on the stability of Ydj1.

In the next step, the functional activity of Ydj1 was investigated. It is well-documented that purified class A J-proteins, such as Ydj1, can prevent protein aggregation even in the absence of Hsp70 (Langer et al., 1992; Schroder et al., 1993). Protein aggregation prevention is a fundamental characteristic of chaperones (Tiroli-Cepeda and Ramos, 2011). Luciferase aggregates at 37°C, and the process can be monitored by an increase in light scattering (turbidity); if a chaperone protects against the aggregation, the scattering decreases (Tiroli and Ramos, 2007). Figure 5B shows that luciferase initiated aggregation after ~17 min at 37°C, as seen by an increase in the light scattering; Ydj1 alone, either before or after the Zn removal process, remained folded. Bovine serum albumin (BSA) was used as a negative control, showing that mixture with any polypeptide is not enough to protect against aggregation. Incubating luciferase with purified Ydj1 resulted in no significant scattering under the conditions tested [Figures 5B,C; 16 ± 4% (*n* = 3)], indicating that the chaperone efficiently protected against aggregation. However, Ydj1 that was subjected to the Zn removal procedure was incapable of protecting luciferase against aggregation, as the scattering light profile was similar to that of luciferase alone [Figures 5B,C; 89 ± 4% (*n* = 3) aggregation]. These results are in good agreement with a site-directed mutagenesis study that was conducted to monitor the effects of the disruption of each ZBD in DnaJ; DnaJ is the *E. coli* class A J-protein that has a ZBD that is structurally conserved with the ZBD of Ydj1. The study found that disrupting ZBDI affects the ability of the protein to protect client proteins against aggregation (Linke et al., 2003). In this study, the action of a DnaJ disrupted ZBDII in protecting luciferase against aggregation was indistinguishable from WT, whereas a DnaJ disrupted ZBDI had no effect on aggregation.

Another study (Fan et al., 2005) also showed the importance of bound Zn for the function of Ydj1. The authors investigated the effect of each ZBD on the function of Ydj1 by disrupting them with site-directed mutagenesis and testing the mutants in complementation assays. They found that an intact ZBDII, but not ZBDI, is required for yeast growth during heat stress. The work also investigated the purified recombinant proteins, showing that both mutants are defective in aiding protein folding. These findings are in good agreement with the results reported in this manuscript as, together, they all point to the importance of Zn bound to Ydj1 for chaperone function, maintenance of homeostasis and, therefore, avoidance of debilitating diseases.

**Figure 6:**
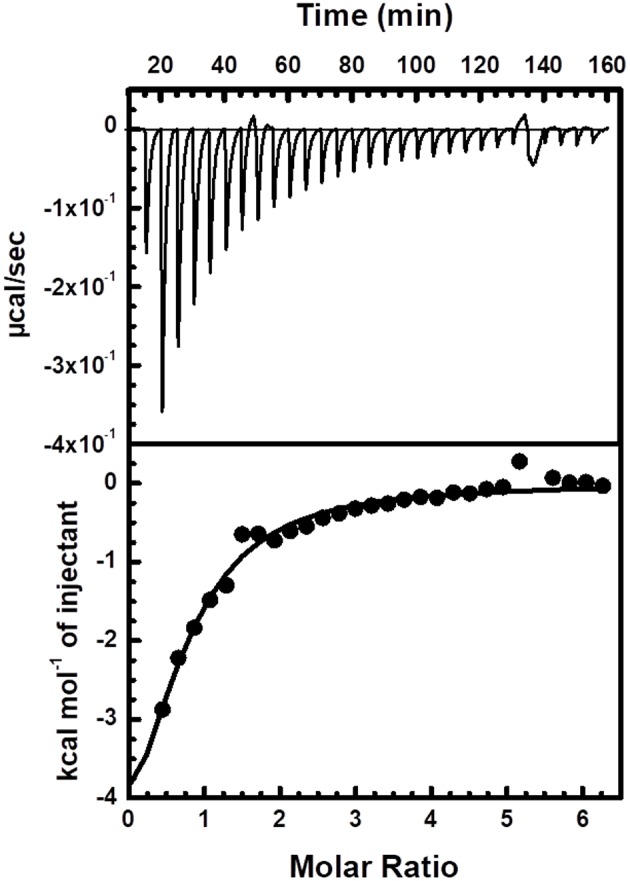
Interaction between Ydj1 and Zn verified by ITC. Thermodynamic parameters were derived from non-linear least-squares fitting implemented by the Origin software. The ITC experiment consisted of 3 μl injections of 3 mM ZnCl_2_ to 30 μM Ydj1 (monomer concentration) with an interval of 300 s between injections. Solutions of titrants and titrates were made from the same buffer [25 mmol L^−1^ Tris-HCl buffer (pH 7.5) and 500 mmol L^−1^ NaCl] and thoroughly degassed prior to use.

Finally, ITC was used to measure the binding of Zn^2+^ to Ydj1 that had been through the zinc removal procedure and the resulting binding isotherms fit well to a one-set-of-sites model (Figure 6 and Figure S2). It is important to point that a higher Ydj1 concentration was not achieved and thus no experiments with increasing protein concentration were performed and that the model only provides a broad understanding of the binding process occurring in solution. In these conditions, the binding reaction was exothermic with ΔH_app_ = −6.9 ± 0.2 kcal mol^−1^, which is measured directly from ITC, and the corresponding *T*Δ*S*_app_ value is of about 3 kcal mol^−1^. Ydj1 was found to bind Zn^2+^ with a K_Dapp_ of 60 ± 15 mM and a stoichiometry of 0.7 ± 0.1 Zn^2+^ per monomer (changing the titrant concentration or repetition generated results with the same order of magnitude as those presented). Although the origin of the low stoichiometry and affinity had not been revealed and may be due to imprecisions in metal:protein binding experiments (Grossoehme et al., 2010), it is clear that the binding is driven by enthalpy. In this case, enthalpy reflects the strength of the interaction between zinc and the protein relative to that between zinc and the solvent (Chao and Fu, 2004). Therefore, the ITC measurements supports the fact that temperature was an important factor in the removal of zinc as discussed above. Additionally, the results also suggest that entropy also has only a small contribution to favor binding indicating that Ydj1 residues involved with zinc coordination undergo little, or none, conformational rigidity upon binding. This observation is supported by the thermal induced unfolding experiments showing only a minor Tm alteration between Ydj1 before and after Zn removal (see above). Of course, although these observations add positively to the area of Ydj1-zinc interaction, more extensive work is necessary to develop a detailed thermodynamic analysis of this binding.

## Conclusion

The proposed methodology in this study provides significant Zn extraction from the Ydj1 co-chaperone, presenting good measures of merit, such as significant precision and accuracy, as well as good limits of detection and quantification. The methodology indicated that recombinant Ydj1 purified from *E. coli* maintains a high affinity for Zn, and all zinc-binding domains kept their structural function. The best results for Zn extraction from Ydj1 were obtained by incubating the co-chaperone (Ydj1) with 1,10-phenanthroline (1 mM) at 37°C and a pH 8.5 for 24 h. This procedure was very effective as the final concentration of Zn was <0.2 mol per mol of Ydj1 monomer. The removal of zinc had no apparent effect on the overall secondary structure of the protein and negligible effect on its stability as measured by thermal-induced unfolding. However, the impact on function was drastic, as Ydj1 lost its chaperone activity and was not capable of protecting a protein from aggregation. This work has a clear potential to aid researchers studying the effects of zinc removal in proteins and to shed light on the importance of this metal to cell homeostasis.

## Author Contributions

JdJ and AA: data collection, analysis, and interpretation. MA and CR: data analysis and interpretation. CR: experimental design. All authors drafted and critically reviewed the article.

### Conflict of Interest Statement

The authors declare that the research was conducted in the absence of any commercial or financial relationships that could be construed as a potential conflict of interest.

## Supplementary Material

Figure S1 Growth tolerance to different Zn concentrations. **(A)** Representative growth of serial dilutions of BY4741 and Δ*ydj1* transformed with empty vector and spotted at different ZnSO_4_ concentrations (as indicated) on SG-U plates. Strains were grown at 30°C for 72 h. See text for details. **(B)** Growth rates from **(A)**. A bar represent the mean with SEM of each condition (performed 3 times in triplicates). BY4741 and Δ*ydj1* are represented by black and gray bars, respectively. The growth of cells treated with each condition was compared to BY4741 behavior without zinc (determined as 100%). Statistical significance is indicated by asterisks (^*^*p* < 0.10; ^**^*p* < 0.05; ^***^*p* < 0.01).

Figure S2 Interaction between Ydj1 and Zn verified by ITC. Thermodynamic parameters were derived from non-linear least-squares fitting implemented by the Origin software. ITC experiment consisted of 3 μl injections of either 2 (left) or 4 (right) mM ZnCl_2_ to 30 μM Ydj1 (monomer concentration) with an interval of 300 s between injections. Solutions of titrants and titrates were made from the same buffer [25 mmol L^−1^ Tris-HCl buffer (pH 7.5) and 500 mmol L^−1^ NaCl] and thoroughly degassed prior to use.
