# Supplementary figures and images for: Optimization of a Methodology for Quantification and Removal of Zinc Gives Insights Into the Effect of This Metal on the Stability and Function of the Zinc-Binding Co-chaperone Ydj1
Source: Front Chem. 2019 Jun 11;7:416. doi: 10.3389/fchem.2019.00416 (PMC6584821; doi:10.3389/fchem.2019.00416)

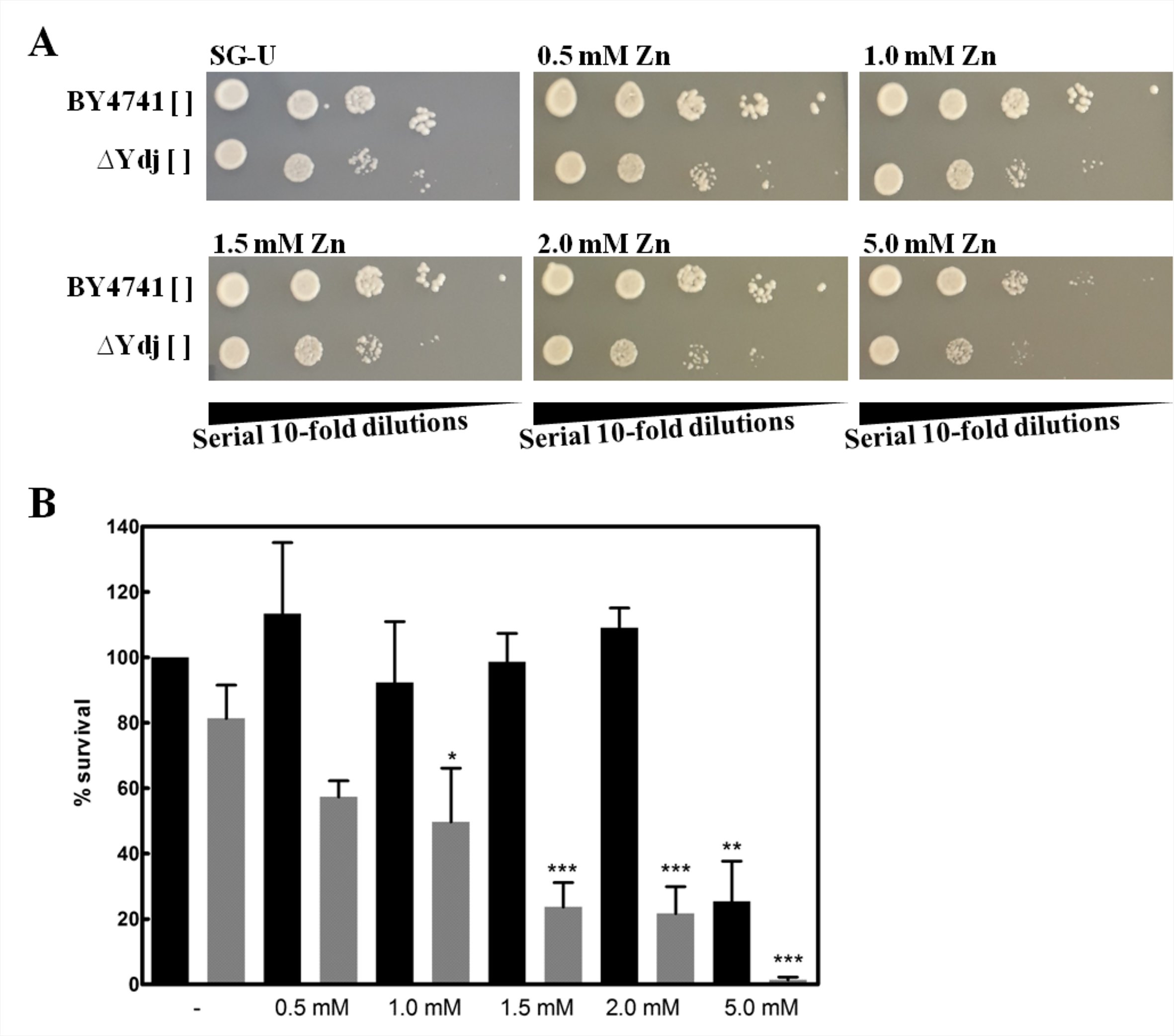

Supplement: Figure S1 — Growth tolerance to different Zn concentrations. (A) Representative growth of serial dilutions of BY4741 and Δydj1 transformed with empty vector and spotted at different ZnSO4 concentrations (as indicated) on SG-U plates. Strains were grown at 30°C for 72 h. See text for details. (B) Growth rates from (A). A bar represent the mean with SEM of each condition (performed 3 times in triplicates). BY4741 and Δydj1 are represented by black and gray bars, respectively. The growth of cells treated with each condition was compared to BY4741 behavior without zinc (determined as 100%). Statistical significance is indicated by asterisks (*p < 0.10; **p < 0.05; ***p < 0.01). [file Image_1.TIF]

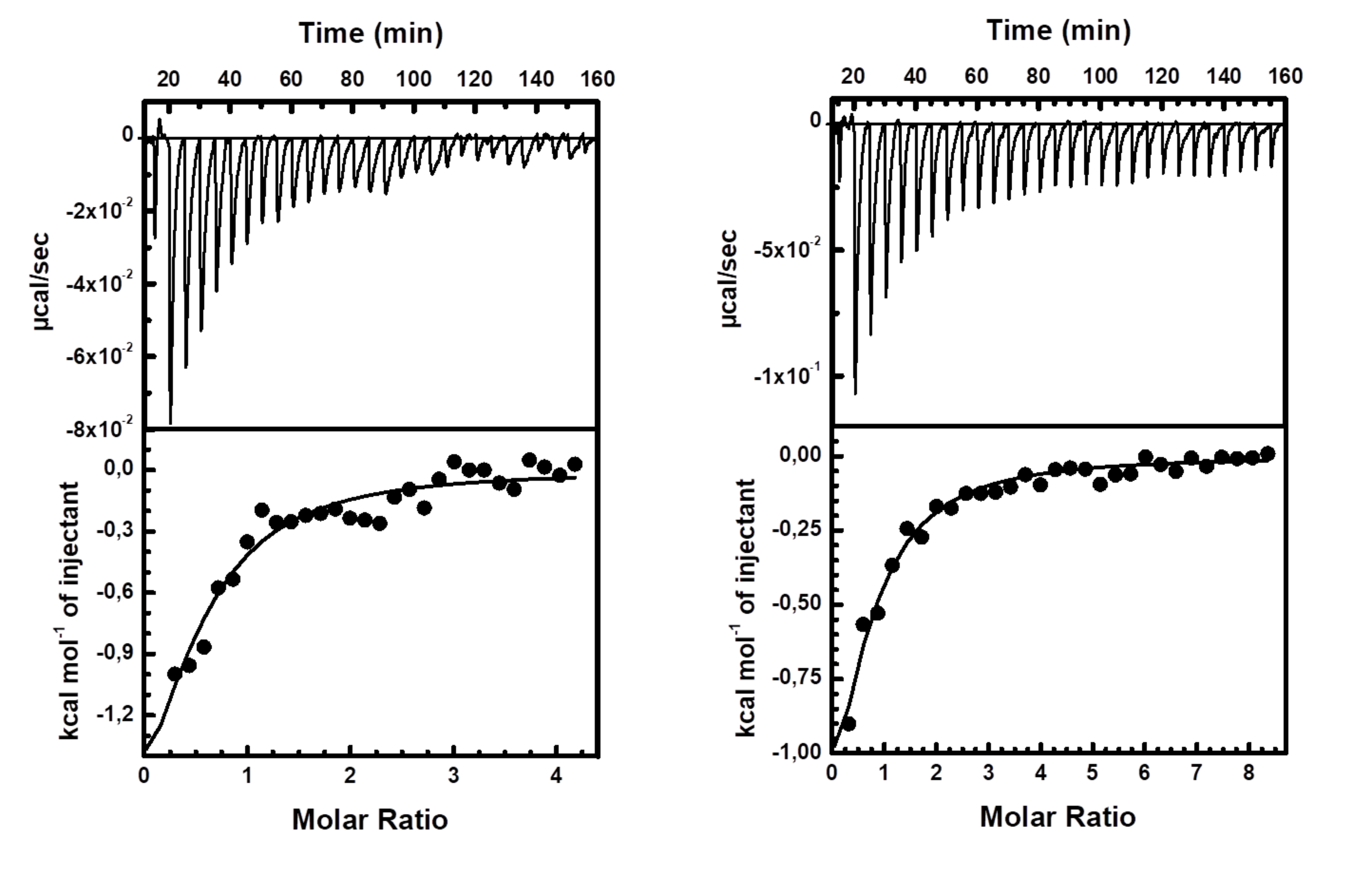

Supplement: Figure S2 — Interaction between Ydj1 and Zn verified by ITC. Thermodynamic parameters were derived from non-linear least-squares fitting implemented by the Origin software. ITC experiment consisted of 3 μl injections of either 2 (left) or 4 (right) mM ZnCl2 to 30 μM Ydj1 (monomer concentration) with an interval of 300 s between injections. Solutions of titrants and titrates were made from the same buffer [25 mmol L−1 Tris-HCl buffer (pH 7.5) and 500 mmol L−1 NaCl] and thoroughly degassed prior to use. [file Image_2.TIF]
